# Basic Accountability to Stop Ill-Treatment (BASI); Study Protocol for a Cluster Randomized Controlled Trial in Rural Tanzania

**DOI:** 10.3389/fpubh.2018.00273

**Published:** 2018-09-24

**Authors:** Redempta Mbatia, Jessica Cohen, Martin Zuakulu, Appolinary Bukuku, Shikha Chandarana, Eliudi Eliakimu, Sisty Moshi, Elysia Larson

**Affiliations:** ^1^Tanzania Health Promotion Support (THPS), Msasani, Tanzania; ^2^Global Health and Population, Harvard T. H. Chan School of Public Health, Boston, MA, United States; ^3^Ministry of Health, Community Development, Gender, Elderly and Children—MoHCDGEC, Dodoma, Tanzania

**Keywords:** patient experience, accountability, report cards, quality of health care, cluster randomized trials

## Abstract

**Background:** Poor health system experiences negatively affect the lives of poor people throughout the world. In East Africa, there is a growing body of evidence of poor quality care that in some cases is so poor that it is disrespectful or abusive. This study will assess whether community feedback through report cards (with and without non-financial rewards) can improve patient experience, which includes aspects of patient dignity, autonomy, confidentiality, communication, timely attention, quality of basic amenities, and social support.

**Methods/Design:** This cluster-randomized controlled study will randomize 75 primary health care facilities in rural Pwani Region, Tanzania to one of three arms: private feedback (intervention), social recognition reward through public reporting (intervention), or no feedback (control). Within both intervention arms, we will give the providers at the study facilities feedback on the quality of patient experience the facility provides (aggregate results from all providers) using data from patient surveys. The quality indicators that we report will address specific experiences, be observable by patients, fall into well-identified domains of patient experience, and be within the realm of action by healthcare providers. For example, we will measure the proportion of patients who report that providers definitely “explained things in a way that was easy to understand.” This feedback will be delivered by a medical doctor to all the providers at the facility in a small group session. A formal discussion guide will be used. Facilities randomized to the social recognition intervention reward arm will have two additional opportunities for social recognition. First, a poster that displays their achieved level of patient experience will be publicly posted at the health facility and village government offices. Second, recognition from senior officials at the local NGO and/or the Ministry of Health will be given to the facility with the best or most-improved patient experience ratings at endline. We will use surveys with parents/guardians of sick children to measure patient experience, and surveys with healthcare providers to assess potential mechanisms of effect.

**Conclusion:** Results from this study will provide evidence for whether, and through what mechanisms, patient reported feedback can affect interpersonal quality of care.

**Pan African Clinical Trials Registry (PACTR):** 201710002649121 Protocol version 7, November 8, 2017

## Introduction

There is growing evidence that patients experience low quality healthcare in low- and middle-income countries (1–3). Providers often lack the knowledge, training and incentives to examine, diagnose, and treat patients effectively. A survey of Tanzanian rural public facilities in 2014 found that providers had low compliance (24.1%) to clinical guidelines for handling maternal and neonatal complications and diagnostic accuracy was only 43.9% (4). Poor quality also extends to interpersonal care: across seven sub-Saharan African countries only 39% of parents/guardians were told about danger signs that they should look for in their sick child (5). A particularly egregious case of poor patient experience is disrespect and abuse: disrespectful treatment during labor and delivery ranges from 12–28% of deliveries in East Africa (6–8) and in one region of rural Tanzania, 14% of women reported disrespectful care during their most recent outpatient care visit for their child (Larson et al., under review).

Poor patient experience is a marker of a poorly functioning health system. There are several consequences, two of which are discussed here. First, disrespectful care violates individuals' fundamental rights to be treated with dignity. Second, data from both low- and high- income countries suggest that interpersonal quality of health care is a primary determinant of patient utilization of future care (9, 10). Experiences and expectations of poor treatment can cause patients and caregivers to avoid or delay seeking care. Poor communication from providers can influence patient adherence to prescribed treatments and to recommended preventive activities (11–13).

Three field experiments in sub-Saharan Africa have found large behavioral improvements with non-financial interventions. In the first, providing feedback reports on health worker performance led to significant improvements in healthcare delivery and health outcomes (14, 15). In the second, social recognition through letters from employers and public interviews, led to improved test scores by health workers (16). In the third, social recognitions through visible charts displaying the public health agents' performance led to increased condom sales (17). Our current study builds on these findings in a new context, assessing whether community feedback through feedback reports, with and without social recognition, can improve patient experience. While providing feedback from patients has been shown to affect provider technical behavior in some contexts, patient feedback has not been extensively explored for patient experience (14, 18–22).

**Figure 1:**
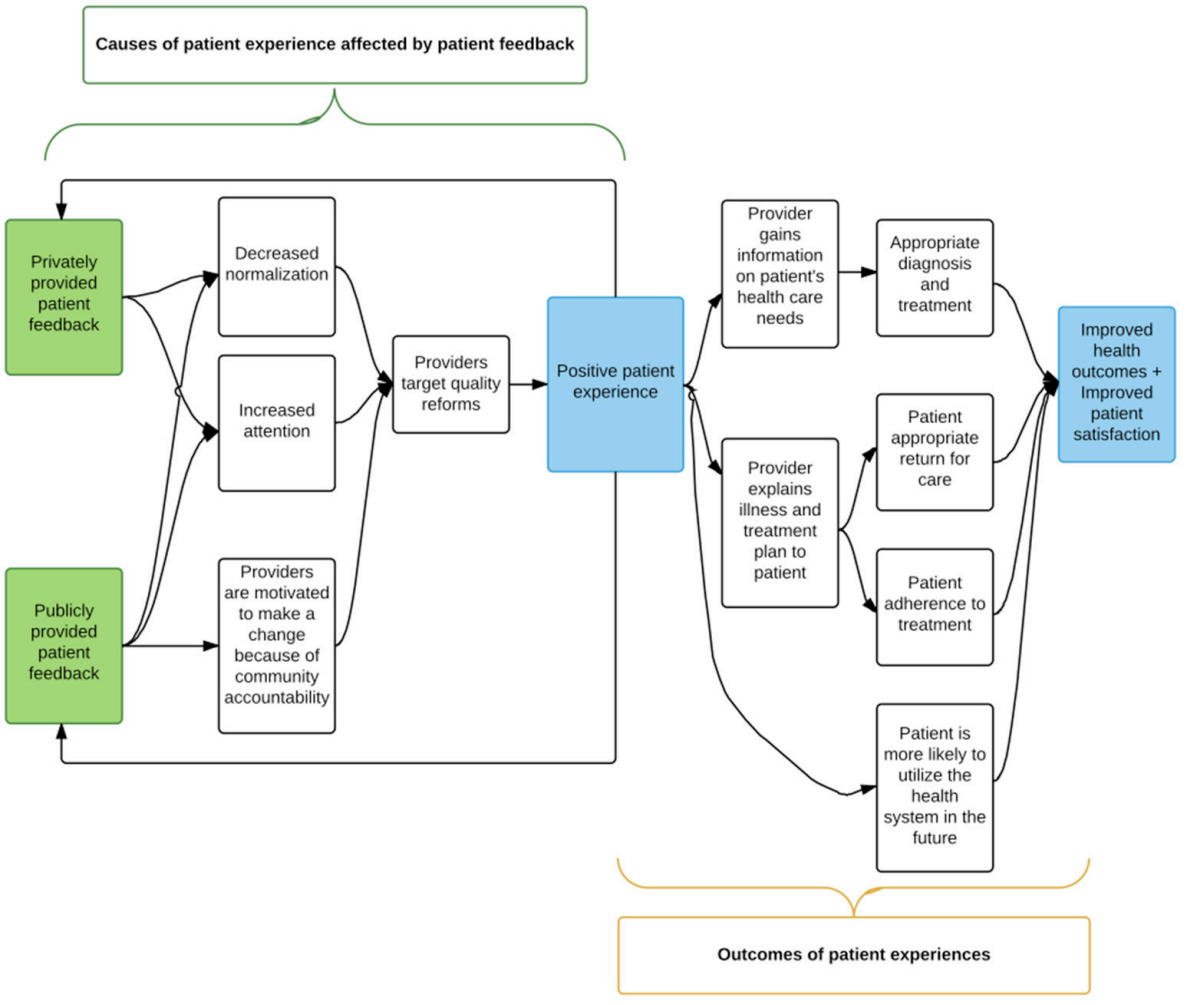
Theory of change for the BASI study.

In this study, we aim to address three causes of poor patient experience: normalization of poor quality, low attention to poor quality, and lack of accountability to the community (Figure 1). Private reports of data on patients' experience serve to increase attention to the problem by giving providers specific information on areas where patients feel they need improvement. By including benchmarks for achievement and discussions with leaders from local organizations, the feedback aims to further decrease normalization of the problem. Social recognition by way of public reporting of providers' success in delivering high quality care, incentivize providers to improve and hold them accountable to the communities they serve. This study will provide evidence for which mechanisms affect patient experience and how communities can be involved to improve quality. The ultimate impact of the study is increased respect and communication during visits for sick children, which is both intrinsically valuable and has been associated with improved health outcomes.

## Methods and analysis

### Study area

The study is being conducted in four rural districts of Pwani Region, Tanzania (Bagamoyo, Kibaha Rural, Kisarawe, and Mkuranga). Sixty-seven percent of the population in the region resides in rural communities, where health services are primarily delivered through dispensaries and health centers. Estimates for literacy rate in the region in 2002 were as low as 50% in rural areas and 69% in urban areas (23). Data from the Demographic and Health Survey indicate that this area is one of the poorer areas in Tanzania, with 53.7% of the population in the bottom two wealth quintiles, and only 1% of the rural Pwani population in the highest wealth quintile (24).

Tanzania Health Promotion Support (THPS), a local non-governmental organization supporting this study, currently supports government-managed health facilities in the study districts. Study facilities are government-managed, THPS-supported, primary care clinics (dispensaries) that had at least 450 sick child visits during the period from October 2016-June 2017. In one district, we included two facilities with fewer than 450 visits in order to have a minimum of 15 facilities per district. We selected primary care clinics (known as dispensaries in Tanzania) for the study, because they are the lowest level of the Tanzanian health system that is expected to provide outpatient care for sick children and are embedded within their communities and thus most likely to be responsive to community feedback (25).

### Study design and methodology

**Figure 2:**
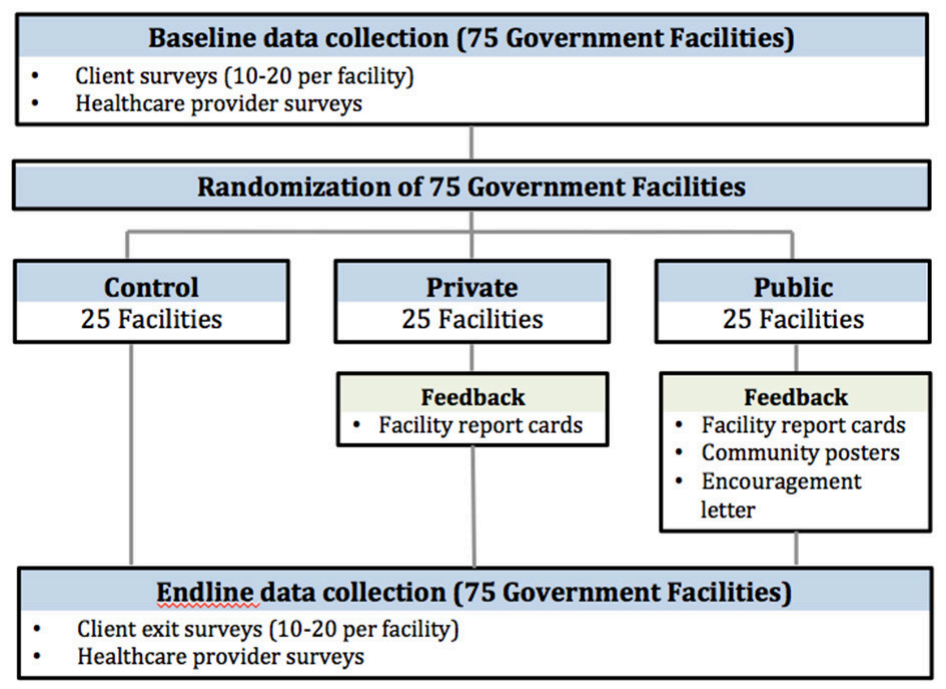
Outline of the BASI study design.

This is a cluster randomized control trial (cRCT) with randomization at the health facility level. The study will begin with baseline data collection, followed by implementation of the intervention in the two study arms, followed by endline data collection (Figure 2). Prior to the start of the study we conducted a small qualitative study to inform the quantitative surveys and intervention materials. We conducted four focus group discussions (FGDs) with groups of four-eight parents/guardians of sick children who visited the health facility in the previous 7 days. We also conducted four FGDs with groups of four-eight healthcare providers. The FGDs took place at five facilities in the study region, in a district separate from the study districts.

The study includes two rounds of data collection, baseline, and endline, at the 75 selected health facilities. We will use two methods of data collection for the parent/guardian surveys. In one district, we will conduct phone interviews. Research assistants will approach parents/guardians after their appointment with the healthcare provider and read a brief recruitment script, which introduces the interviewer, states the main aim of the study, describes why they are being recruited to participate in this survey (i.e., the eligibility criteria), and assurance of confidentiality. If the participants are eligible and agree to participate in the interview 2–7 days later, the research assistants will obtain written consent at the time that their phone number is collected and then follow-up with a phone call. Written consent gives a detailed description of the study, including their rights as a participant. For participants who do not have a phone number to give, we will provide our study phone number and encourage them to “beep” us from any number they have access to in the following 2–7 days. When we receive phone calls, we will return them to complete the survey. In the remaining three districts, parent/guardian surveys will be collected as exit surveys. For exit surveys, research assistants will approach caregivers after their visit with the healthcare provider and inform them of the study. If they express interest, the research assistant will invite them to a private space away from the health facility to further inform them of the study using a recruitment script as described above. A full informed consent will be obtained prior to participation in the study.

At both baseline and endline we will also interview healthcare providers at the study facilities. They will be invited to participate in a structured interview that will include questions on socio-demographics, their job satisfaction, and their opinions on quality of care including a free list exercise in conjunction with a discrete choice experiment. Healthcare providers will be informed that their facility is participating in a study and that are being invited to participate in a survey that will help researchers learn about people's experience with healthcare and how providing feedback to health workers can help them better serve their communities.

Baseline results will be delivered to intervention facilities after all baseline data collection is complete. Approximately 4–7 weeks after the baseline results have been delivered to the intervention facilities, we will conduct the endline surveys. These will be collected from all study facilities in the same format as outlined above (phone surveys in one district and exit surveys in in three districts). In addition, at endline, in order to measure the effect of negative patient experiences on health outcomes and patient behavior, we will interview a subset of parents/guardians by phone ~1–2 weeks after their primary visit. We will ask them about the health of the study child and additional questions about recent illness among their children and their care-seeking behaviors. However, this study is a proof of concept to detect changes in provider behavior and is not necessarily powered to detect differences in these secondary outcomes. Participants for the follow-up survey will be randomly selected from those who complete the endline parent/guardian survey.

The populations living in the study districts are low-literacy and economically disadvantaged as a whole. We therefore expect that study participants will also be low-literacy and economically disadvantaged. All survey materials are written in language that is understandable to low-literacy populations in the local language (Swahili), and were pilot tested for clarity. The surveys will all be conducted by Swahili-speaking Tanzanian research assistants who have experience working with similar populations. The qualitative data collection conducted prior to the start of the cRCT focused on ensuring that survey questions were understandable to this population. All data will be collected using hand-held tablets with the SurveyCTO software. Data will be encrypted to protect confidentiality. All research assistants will undergo training in data collection methods, including privacy, and confidentiality of participants and their data.

### Randomization

After baseline data collection, we will stratify study facilities by district. Within each stratum, we will randomize the facilities to one of three arms: private feedback (intervention), social recognition reward (intervention), or no feedback (control) with a 1:1:1 allocation ratio. The principal investigator will conduct randomization using a random number generator in Stata version 14.2. Parents/guardians will be recruited from the health facilities that they visit and assigned that facility's intervention status. Healthcare providers will likewise be assigned the intervention status of the facility where they work. Participants will not be blinded to the allocation. It is possible that healthcare providers in the control group, or the private feedback group, will learn about the interventions provided in the other arms. This is however unlikely given the short duration of the study. If they do learn of the interventions in the other arms and this causes them to improve the care they provide, this could lead to a dilution of effect.

### Intervention

The study facilities will be randomized into either the control arm or one of two intervention arms: private feedback or public feedback with non-financial reward (social recognition arm). The control arm will undergo the same data collection activities as the intervention arm, but will not be provided with feedback. If there are changes in patient experience due solely to the act of collecting data, those changes will be captured by this control group.

Within both intervention arms we will give the providers at the study facilities feedback on the quality of patient experience the facility provides (aggregate results from all providers) using data from patient surveys. Feedback will be presented as private feedback reports designed using extensive experience from the U.S. and developing countries (26–28). The feedback reports will provide specific information to make clear which provider behaviors should be changed and will include benchmarks for each indicator. Social comparison will be avoided, as this can lead to reduced performance, especially among low-ability individuals (16, 29). The indicators will address specific experiences, be observable by patients, fall into well-identified domains of patient experience, and be within the realm of action by healthcare providers (16, 26, 28–30). For example, we will measure the proportion of patients who report that providers definitely “explained things in a way that was easy to understand.” Feedback will be delivered by a medical doctor to all the providers at the facility in a small group session. A formal discussion guide will be used.

Facilities randomized to the social recognition reward arm have two additional opportunities for social recognition. First, we will create a poster displaying their achieved level of patient experience and post it at the health facility and village executive officer (village leader's) office. This poster will contain the results from five questions on the survey: overall quality, wait time, respect, and greeting. For each item there will be a picture with five possible starts—five representing the best score on the question and zero representing the worst score. The poster will explain the facility's score and the meaning of each individual indicator in Swahili.

The posters are meant to provide public feedback on the patient experience provided at this facility. However, because they contain information that the providers will see as well, this poster will also be privately given to the healthcare providers at the private feedback facilities, but in the instance of private feedback facilities it will not be posted for the community to see. This will allow the providers to understand how to interpret their results, similar to the providers in social recognition intervention arm, limiting the difference between the intervention arms to the public display of the results (and not the content of the information available to the providers).

Second, facilities in the social recognition arm will receive an encouragement design in the format of recognition from senior officials at the local NGO and/or the Ministry of Health. Providers will be told that the two facilities that score the highest on patient experience indicators and the two facilities that show the most improvement on patient experience indicators at the next round of data collection will receive a letter of recognition from the local NGO and/or the Ministry of Health. Evidence suggests that letters of recognition act as social rewards and lead to improved performance (16).

### Participants and selection criteria

This study involves two groups of participants: parents/guardians of sick children and healthcare providers. Parents/guardians are eligible for inclusion in the study if they are accompanying children under 13 years of age for a sick child outpatient visit at a study facility on a day when we are recruiting patients; are 15 years of age or older; and provides informed consent or assent in the case of parents/guardians aged 15–17. For individuals who are 15–17 years old we will ask if they are either the parent or legal guardian of the child to ensure that they are emancipated minors. For individuals older than 18, all adults who accompanied the child, regardless of their relationship to the child are invited to participate. The relationship to the study child is determined during the survey. For a subset of parents/guardians we will invite them to participate in a follow-up phone survey ~1–2 weeks after their initial facility visit.

All skilled healthcare providers in the study facilities will be invited to participate in a structured interview that assesses their views on healthcare quality as well as their job satisfaction. A healthcare provider is eligible for inclusion in the study if s/he is working in a study facility; is a skilled provider (i.e., cadre is nurse, clinical officer, medical officer, or medical doctor); provides outpatient care for sick children; is 18 years of age or older; and provides informed consent.

### Sample size estimation

Our study sample will include 75 government-managed primary health facilities (25 facilities per arm). Our sample size calculations used data from the MNH+ study conducted in the same region in 2016 among 2,002 individuals at 12 health facilities as well as from the Tanzania Demographic and Health Survey 2015–16 (Larson et al., under review) (24). We conducted our sample size calculations based on two indicators of patient experience: disrespectful care and provider communication of child's diagnosis, using baseline prevalence and intraclass correlation coefficients (ICC) determined from the prior studies.

For each of the outcomes of interest we calculated the minimum detectable difference given alpha = 0.05, beta = 0.20, 25 clusters per arm, and 15 individuals per cluster. We calculated the detectable difference assuming a difference in differences design with baseline correlation of 0.8. All calculations use the *clustersampsi* command in Stata 14.1. We obtained a sample size of 1,125 parents/guardians at baseline and endline. Our sample is powered to detect a difference in difference of disrespectful care of 5 percentage points (from 14% at baseline to 9% at endline) or a difference in difference in communication of the child's diagnosis of 8 percentage points (from 46 to 54%).

### Measures

The primary outcome of interest is patient experience. To develop measures of patient experience we drew from the literature on respectful maternal care (31), health systems responsiveness (32, 33), and patient experience (34). The final survey questions include previously used measures of disrespectful care in Tanzania and international surveys of patient experience conducted in sub-Saharan Africa by the World Health Survey and the Service Provision Assessment (10, 33, 35, 36). We also take advantage of publically available and widely used measures from the U.S (30). These indicators are refined and expanded based on our qualitative work. Patient experience includes aspects of patient dignity, autonomy, confidentiality, communication, timely attention, and social support (32, 37). Composite indicators of patient experience will be developed from the individual indicators in order to represent the main sub-domains, specifically for effective communication and respectful care.

Secondary outcomes are measured through the parent/guardian survey, the parent/guardian follow-up survey, and the healthcare provider surveys. From the parents/guardians we will measure their utilization behavior, rating of technical quality, such as the providers' medical knowledge, satisfaction with care, likelihood of recommending the health facility, rating of overall quality, child's illness resolution, and confidence in the health system. From the healthcare providers, we measure attentiveness to patient experience, value of patient experience, perceptions of the quality of care they provide, job satisfaction, stated motivation, and attrition.

### Analysis

The primary causal identification strategy for the intent to treat analysis will be a difference-in-differences (DID) analysis comparing change in outcomes in the intervention groups to changes in outcomes in the control group. DID analyses have the advantage of controlling for differences between the groups at baseline. We will use the following model:

$$\begin{array}{l} Y_{if}=\;\mu +\;\gamma \cdot Private_{if}+\lambda \cdot T_{if}+\;\delta \cdot (Private_{if}\cdot T_{if})\\ \;+\eta \cdot Reward_{if}+\nu \cdot (Reward_{if}\cdot T_{if})+\varepsilon_{if} \end{array}$$

Here, *Y*_*if*_ is the outcome for individual *i* in facility *f*, μ is the mean outcome among individuals in the control facilities at baseline, γ is the mean difference between individuals in the intervention groups and control group at baseline, λ is the effect of time (T is a post intervention indicator), and δ and η are the estimators of interest: the difference between the change in the private or reward intervention groups and the change in the control group, respectively. ε_*if*_ is the individual error clustered at the facility level. We will conduct a sensitivity analysis assessing endline differences only and one imputing missing data. Additional secondary analyses will use discrete choice data and free-list responses from the providers to assess their views on quality of care. These analyses will allow us to assess if providers are inattentive to patient experiences or if they do not think that they are important, and if this changes as a result of the intervention.

## Discussion and implications

The study will assess three causes of poor patient experience: normalization of poor quality, low attention to poor quality, and lack of accountability to the community. Private reports of data on patients' experiences serve to improve providers' awareness of problems by giving providers specific information on areas where patients feel they need improvement. Social recognition by way of public reporting of providers' success in delivering high quality care, incentivize providers to improve and hold them accountable to the communities they serve. This study will provide evidence for which mechanisms affect patient experience and how communities can be involved to improve quality.

If successful, the intervention will provide a method for improving patient experience during outpatient care for sick children. The study will also provide a validated tool for measuring patient experience that could be used in similar contexts, contributing to the body of knowledge on how to measure the quality of care and in particular on the principles to adhere to when measuring quality of care (38). After completion of the study and analysis of the results, we intend to thoroughly discuss the anticipated mechanism of effect, the evidence that supports or contradicts these mechanisms. We will discuss how these findings fit into the literature on both the measurement of patient experience, and the accountability of healthcare providers to delivering high quality interpersonal care.

We expect the study to inform the Ministry of Health, Community Development, Gender, Elderly and Children; Regional and Council Health Management Teams; as well as local NGOs on sustainable and efficient means of addressing gaps related to patient experience as contributors to quality care. The current Health Sector Strategic Plan IV 2015 –June 2020 (HSSP IV) includes plans to promote and strengthen relationships between communities and health facilities, including through empowerment and accountability of health facility governing committees (25). Tested models for achieving these goals are of great interest to policy makers, both in Tanzania and countries with similar gaps in quality of care.

### Limitations

There are several potential limitations to this study. First, it is possible that healthcare providers could ask patients to give a favorable report of their experience. While there is no incentive for providers to ask patients to misreport the care received in the control and private feedback arms, there is some concern that providers may ask patients to misreport their care in the reward arm. However, even in this arm the incentive is low, as there is no punishment for poor care and no comparison to other providers. We will ask respondents whether a healthcare provider spoke to them about giving a positive response to the survey. Second, it is possible that healthcare providers could drop out of the study, or leave the health facility and no longer be eligible, between baseline and endline. We will track healthcare providers to determine if they decline to participate in follow-up (drop out) or leave the health facility. Third, because we are not providing feedback to specific providers on their individual behavior, but rather to a group of providers on the aggregate behavior at the health facility, that healthcare providers will not feel that they as an individual need to change. However, it is also possible that they will try to influence their colleagues' behavior as well as their own. We will use the provider surveys to capture information on their own feelings toward patient experience to build a theory for how the intervention did, or did not, affect change. Finally, the results of this study may not be generalizable to different levels of health facilities (e.g., care provided at hospitals), different types of care (e.g., care provided to adults), or different contexts outside of rural Tanzania. This is a proof of concept study and if the intervention is successful in improving provider behavior, the concept will need to be studied in other contexts.

## Ethics and dissemination

This study was reviewed and approved by the ethics committees at the Harvard T. H. Chan School of Public Health in the United States and the National Institute for Medical Research in Tanzania prior to the start of data collection. Written or oral informed consent will be obtained by Swahili-speaking, Tanzanian research assistants from all participants prior to participating in the study, and participants will be reminded that they can withdraw at any time. All spontaneously reported adverse events and other unintended effects of the study will be collected, assessed, and managed by the principal investigator. Adverse events will be reported to the ethics committees. Any updates to the study will go through ethics committee approval and will be included in the PACTR.

We have a two-part dissemination strategy. After all data have been collected and the results have been analyzed, we will develop a policy report to be shared with all stakeholders, including the district, regional, and national ministries of health. We will also develop and submit academic manuscripts to peer-reviewed journals to share our results more widely with the academic, policy-making, and implementation communities.

## Availability of data and material

Data sharing is not applicable to this article as no datasets were generated or analysed during the current study. The final trial dataset will be accessible by the principal investigator and co-investigators. A minimal, de-identified dataset will be made more widely available within two years of study completion.

## Author contributions

RM and EE advised on the study design and intervention and provided contextual feedback. RM, SC, and EL prepared the first draft. EL conceived the research questions, designed the study, and conducted data analysis. JC advised on the study design, intervention, and research methods. MZ supervised data collection. AB and SM supervised the intervention. All authors read and approved the final manuscript.

### Conflict of interest statement

The authors declare that the research was conducted in the absence of any commercial or financial relationships that could be construed as a potential conflict of interest.
